# Male pseudohermaphroditism: A case study of 46,XY disorder of sexual development using whole‐exome sequencing

**DOI:** 10.1002/ccr3.3286

**Published:** 2020-09-10

**Authors:** Oxana Yu. Naumova, Sergey Yu. Rychkov, Olga V. Burenkova, Maria Yu. Solodunova, Irina V. Polyanskaya, Irina A. Arintcina, Marina A. Zhukova, Irina V. Ovchinnikova, Olga V. Zhukova, Elena L. Grigorenko

**Affiliations:** ^1^ Vavilov Institute of General Genetics Russian Academy of Sciences Moscow Russia; ^2^ Saint Petersburg State University Saint Petersburg Russia; ^3^ University of Houston Houston TX USA; ^4^ Specialized Neuropsychiatric Baby Home #13 Saint Petersburg Russia; ^5^ Baylor College of Medicine Houston TX USA

**Keywords:** 46, XY disorder of sexual development, androgen insensitivity syndrome, disease‐related variant, whole‐exome sequencing

## Abstract

The study shows that whole‐exome sequencing is a promising approach to detect novel variants—and gene candidates in DSD, that, as a future direction, may improve the diagnostic gene panels for this heterogeneous disorder.

## Graphical abstract

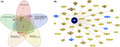

## 1 INTRODUCTION

This study, utilizing whole‐exome sequencing (WES), reports on a previously detected disease‐related variant in the androgen receptor gene *AR* [c.528C>A (p.Ser176Arg)] and novel candidate variants in the *DHCR24*, *BMPR1B*, *NODAL*, and *WDR48* genes detected in the genome of a 15‐month‐old child diagnosed with MPH, manifested as partial androgen insensitivity syndrome (AIS).

46,XY disorder of sexual development (DSD)—or male pseudohermaphroditism according to formerly used nomenclature—is a condition defined by the presence of female‐like or incompletely differentiated external genitalia in an individual with a Y chromosome. It is the most diverse type of DSD concerning to both clinical manifestations and etiology. The molecular etiology of 46,XY DSD is almost always attributed to genetic lesions that might be delineated as cytogenic alterations and gene mutations.^1^ The former is usually manifested as 45,X/46,XY mosaicism, while the latter as mutations that disrupt either the production of androgens or tissue response to androgens.

Androgen resistance is one of the most common causes of 46,XY DSD; it is manifested as AIS and classified based on clinical phenotypes into three categories: complete (CAIS), partial (PAIS), and mild (MAIS) forms. In most cases, this is an X‐linked disease due to alterations to the androgen receptor gene *AR*, whose variety of damaging effects may partly determine the severity of AIS.^2^, ^3^ Besides, AIS may also be related to a deficit in AR coactivators caused by mutations in the relevant genes, such as *NR5A1* encoding the steroidogenic factor‐1 protein,^4^ which among other functions promotes the transcription of steroidogenic enzymes and steroidal receptors. Other possible molecular causes of the DSD are related to deficiencies in key enzymes controlling the androgens' production associated with various deleterious variants in the relevant genes: Cytochrome P450 17α‐hydroxylase (*CYP17A1*) and 17β‐hydroxysteroid dehydrogenase (*HSD17B3*), essential for steroidogenesis, and 5α‐reductases (*SRD5A1* and *SRD5A2*), necessary for the conversion of testosterone into its more potent form, dihydrotestosterone.^5^, ^6^, ^7^, ^8^ These deficiencies may manifest in phenotypes identical to CAIS or severe PAIS.

To date, dozens of candidate genes have been identified for DSD.^9^ Besides, a variety of mutational events (and their combinations) in genes involved in sexual differentiation and development may cause DSD and lead to congenital conditions that fall under the broad classification of DSD. Herein, we report on exonic variants detected in the genome of a toddler diagnosed with PAIS, which might be causative for the clinical phenotype expression.

## 2 CASE PRESENTATION

A 15‐month‐old child of mixed Slavic‐Turkic descent residing in a state‐run orphanage in the city of Saint Petersburg, Russia, was recruited for this study. Written informed consent for the child examination and genetic analysis was obtained from the orphanage administrators. The Saint Petersburg University Research Board approved the study.

The participant was the first child of a 25‐year‐old woman of Uzbek ethnicity (Central Asia), born after a 35‐week pregnancy. According to the medical records, the delivery was complicated; the child had birth injuries, including intracranial laceration and hemorrhage. His characteristics at birth were as follows: a 5‐minute Apgar score of 7, weight 2450 g, height 47 cm, and chest and head circumferences, respectively, 32 and 30 cm. The newborn had congenital conditions that fall under the DSD clinical phenotype. His gonadal (testicles) and genetic (46,XY) sex was male while the external genitalia were ambiguous: micropenis, penoscrotal hypospadias, perineal meatus, ventral preputial split, and testicular hypoplasia. Testosterone levels were undetectable, but showed a response to the human chorionic gonadotrophin (hCG) stimulation with testosterone/dihydrotestosterone (T/DHT) ratio of 7 and testosterone/androstenedione (T/A) ratio of 5, at the age of 15 months. Trial of short‐course hormonal therapy (Omnadren) was prescribed that resulted in an increase in the size of the penis, scrotal skin, and foreskin; no other treatment was administered. The child was diagnosed with PAIS.

In addition to PAIS, this 15‐month‐old child had mixed specific developmental disorders, congenital malformations of the eyes, hypermetropic astigmatism, and frequent acute respiratory infections. Physical examination and comparison of his growth characteristics with the WHO Child Growth Standards revealed a low height‐for‐age (*z* = −1.24; 10.70%) and a head circumference‐for‐age that correspond to the parameters of microcephaly (*z* = −2.15; 1.59%). Behavioral testing showed a delay in gross and fine motor development according to the Mullen Scales of Early Learning, MSEL^10^ (the 1st percentile) and a delay in lexical development measured using the MacArthur‐Bates Communicative Development Inventory, MB‐CDI ^11^ (3 SD below the mean at age; *z* = −3.54).

## 3 DETECTION OF EXONIC CANDIDATE VARIANTS

DNA was isolated from peripheral blood using the FlexiGene DNA Kit (Qiagen) according to the manufacturer's instructions. WES was conducted using the Ion Torrent platform following the manufacturer's instructions (Thermo Fisher Sci): exome enrichment and library preparation were performed using the Ion AmpliSeq Exome RDY Kit, and the WES library was sequenced using the Ion GeneStudio S5. The sequence output data were 5.9Gb, 31M mapped reads, and 94.8 mean depth. WES read alignment and variant calling were accomplished using the Torrent Suite Software and Variant Caller plug‐in. The data on variant calls that support the findings of this study are available from the corresponding authors upon reasonable request.

**FIGURE 1:**
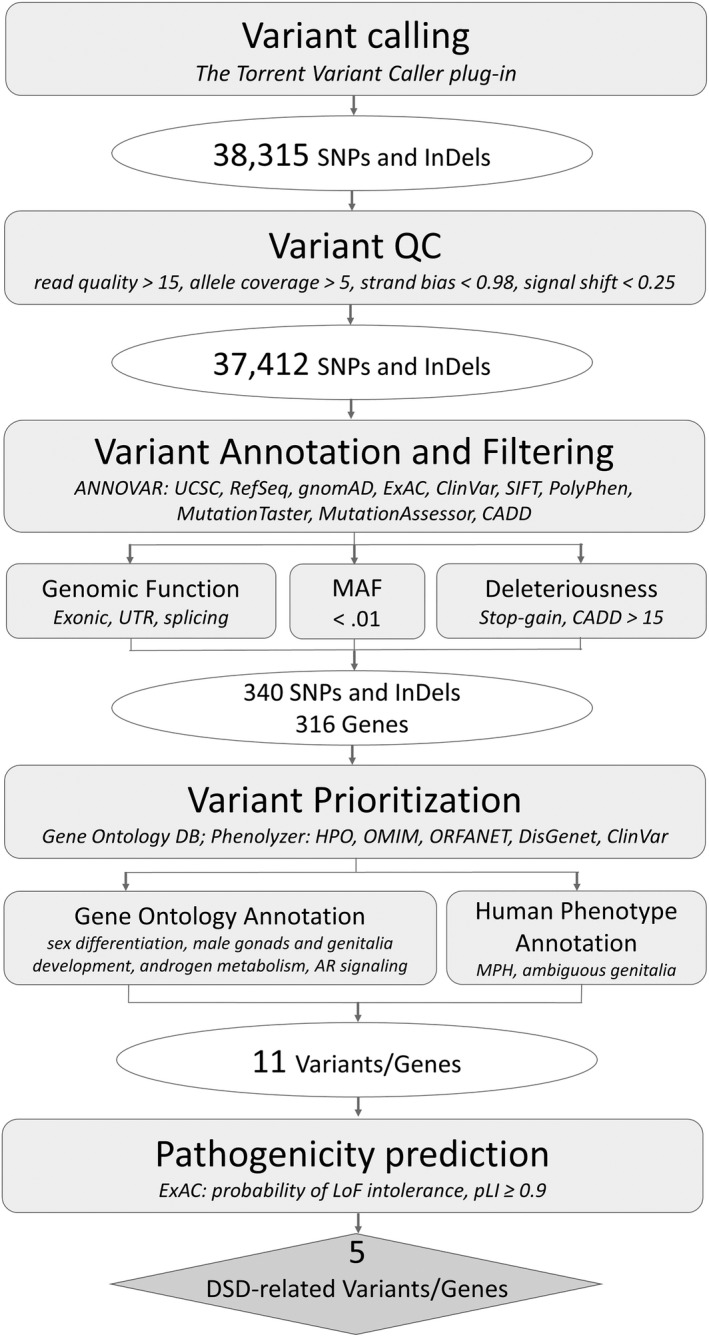
The workflow of the exonic variant reduction and prioritization procedure used to identify rare disease‐related variants in the genome of the child diagnosed with 46,XY DSD

Variant functional annotation was performed using the ANNOVAR (ANNOtate VARiation) bioinformatics tool for the interpretation and prioritization of genomic variants.^12^ Variants were examined by filtering them against known variation databases; the inclusion criteria were as follows: the localization within an exon, UTR (UnTranslated Region flanking a coding sequence on RNA) or splice site; a low frequency in the general population—minor allele frequency, MAF < 0.01 according to the Exome Aggregation Consortium (ExAC) ^13^ and the Genome Aggregation Database (gnomAD) ^14^; and a deleterious effect on protein function—the Combined Annotation‐Dependent Depletion (CADD) ^15^ deleteriousness prediction score >15 (Table S1). Variants were prioritized by filtering the genes containing a rare deleterious variant against the Gene Ontologies (GO)^16^ related to sexual development (Table S2), and by ranking these genes with regard to prior knowledge on the associations with DSD and DSD‐related Human Phenotype Ontologies^17^ (HPO), using the Phenolyzer ^18^ (Table S3). An overview of the variant reduction and prioritization procedure is illustrated in Figure 1.

**FIGURE 2:**
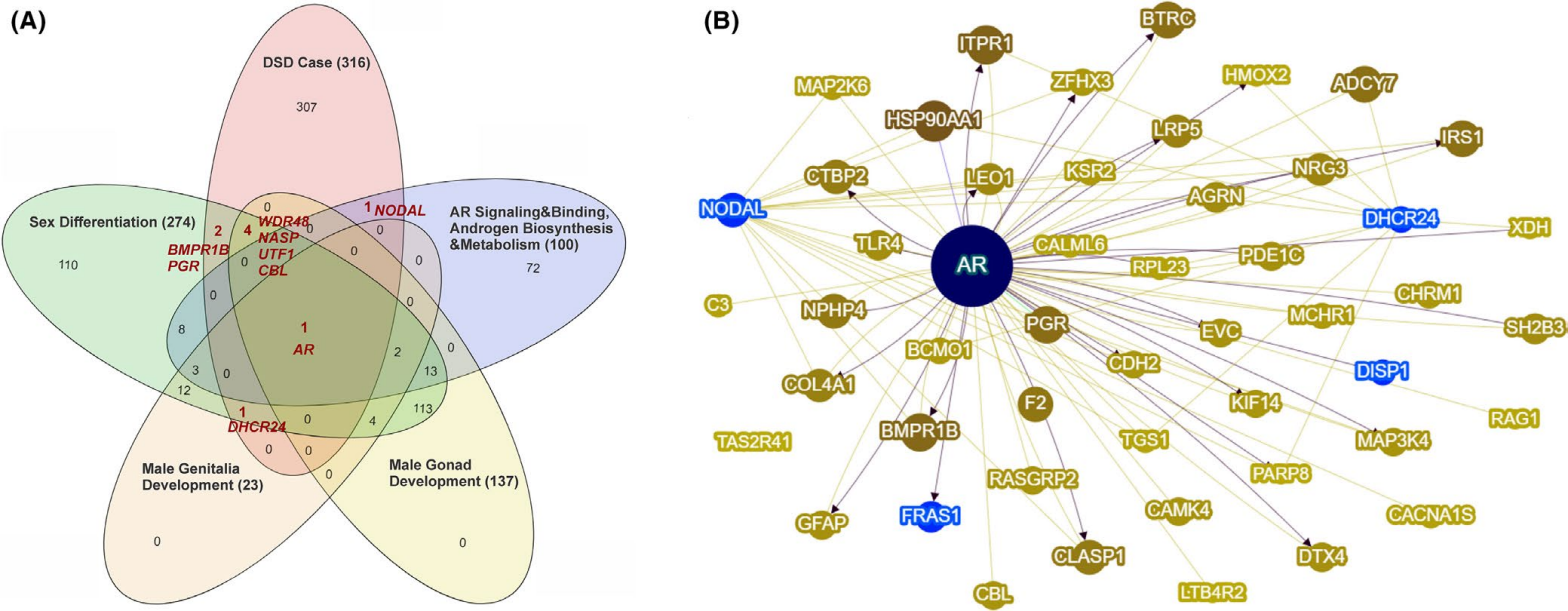
Venn diagram (A) represents the distribution of 46,XY DSD candidate genes containing a rare deleterious variant in the child's genome (red circle of the diagram) with regard to genes' annotation to a GO related to sex development. Each GO term represented as a circle; numbers show the number of genes; and the total number of genes assigned to a GO is shown in brackets. The intersect areas with the DSD case category highlight nine genes involved in sex differentiation processes and androgens' metabolic and signaling pathways, which have deleterious variants in the child's genome. The Phenolyzer gene network (B) prioritizes five candidate genes (marked in black) for DSD and/or ambiguous genitalia phenotype; the size of a node represents the Phenolyzer' score of the gene‐phenotype association; the edges represent gene interactions; and transcriptional regulation is shown by arrows

In the child's exome, we detected 340 rare deleterious variants in 316 genes (Figure 1); the list of these variants represented in Table S1. Among those 340, nine mutations occurred in genes involved in sex differentiation and development: seven heterozygous missense mutations (or mutations resulting in amino acid substitutions in the protein encoded) in *DHCR24*, *NODAL*, *BMPR1B*, *PGR*, *NASP*, *UTF1*, and *CBL*; a stopgain microdeletion, resulting in premature termination of the protein translation, in *WDR48*; and a hemizygous missense mutation in *AR* (Figure 2A; Table 1). Phenotype‐centered gene prioritization by the Phenolyzer revealed two additional candidates: missense mutations in *FRAS1* and *DISP1* known to be associated with Fraser syndrome—a genetic disorder characterized by multiple abnormalities including genital malformations and dysfunctions (Figure 2B; Table 1).

**TABLE 1:** Eleven rare deleterious exonic variants that affect genes involved in sex differentiation and development processes, and may be associated with the clinical manifestations of DSD

Variant					Gene				Association with relevant phenotypes^c^
dbSNP	Position	Change	Function	Zygosity	Name	Function	ExAC pLI score^a^	Phenolyzer score^b^	
rs777131133	chr0X:66765516	C>A	Missense	Hemi	*AR* ^d^	Androgen receptor	0.9949	0.5984	46,XY DSD, androgen insensitivity syndrome, ambiguous genitalia
N/A	chr01:55349314	G>T	Missense	Hetero	*DHCR24* ^d^	24‐dehydrocholesterol reductase	0.9691	0.0770	Ambiguous genitalia, desmosterolosis
rs778607015	chr10:72195653	G>A	Missense	Hetero	*NODAL* ^d^	Nodal growth differentiation factor	0.9472	0.0710	Ambiguous genitalia
N/A	chr03:39136143	TG(del)	Stopgain	Homo	*WDR48* ^d^	WD repeat domain 48	0.9998	N/A	N/A
rs190883013	chr04:96025637	C>T	Missense	Hetero	*BMPR1B* ^d^	Bone morphogenetic protein receptor type 1B	0.9917	N/A	N/A
rs11571143	chr11:100999654	C>T	Missense	Hetero	*PGR*	Progesterone receptor	0.7848	N/A	N/A
rs28365868	chr01:46049849	G>C	Missense	Hetero	*NASP*	Nuclear autoantigenic sperm protein	0.4702	N/A	N/A
rs529391166	chr10:135044882	C>T	Missense	Hetero	*UTF1*	Undifferentiated embryonic cell transcription factor 1	0.1508	N/A	N/A
rs2227988	chr11:119156193	C>T	Missense	Hetero	*CBL*	Cbl proto‐oncogene	0.0068	N/A	N/A
rs768744785	chr4:79410160	G>A	Missense	Hetero	*FRAS1*	Fraser extracellular matrix complex subunit 1	0.0000	0.0999	Fraser syndrome, ambiguous genitalia
rs148437031	chr1:223177006	G>A	Missense	Hetero	*DISP1*	Dispatched RND transporter family member 1	0.0000	0.0710	ambiguous genitalia

^a^ The ExAC pLI score is the probability of a gene to be LoF intolerant ^13^: a pLI ≤ 0.1 indicates the extreme LoF tolerance of a gene, a pLI close to 0.5 implies heterozygous LoF tolerance, and a pLI ≥ 0.9 indicates the high probability of gene haploinsufficiency.

^b^ The normalized rank score of phenotype‐based prioritization of candidate genes is presented.

^c^ The associations with relevant phenotypes of DSD and ambiguous genitalia are given based on the results of the Phenolyzer' annotation against the OMIM, HPO, ClinVar, Orphanet, and DisGenNET databases.

^d^ Genes identified as candidate genes for DSD in the studied case.

Altogether, we obtained a final list of 11 variants potentially responsible for the DSD phenotype (Table 1). Regarding the pathogenicity of these variants, six of them may be considered “neutral” according to a low probability of the gene to be loss‐of‐function (LoF) intolerant, based on the ExAC pLI score.^13^ Hence, the remaining five variants with the highest scores—a pLI ≥ 0.9 indicating a high probability of the gene haploinsufficiency—were defined as disease‐related variants located in the *AR, DHCR24*, *NODAL*, *BMPR1B*, and *WDR48* genes.

## 4 DISCUSSION

Both of the gene‐prioritization algorithms we applied in this study indicated a key role of androgen receptor (*AR*) alteration in the manifestation of abnormalities in sexual development in the studied case (Figure 2). To date, about 400 *AR* variants have been reported in public repositories, and about 60 of them have been associated with AIS.^3^, ^19^ This list of AIS‐related variants does not contain the variant we found in the DSD case (rs777131133; Table 1). However, this variant has been reported in a study of targeted sequencing of genes involved in sexual development in a cohort of 70 46,XY DSD patients from China and has been defined by the authors as a diagnostic disease‐related variant.^9^ Two of 70 patients with phenotypes similar to the DSD case presented here (micropenis, penoscrotal hypospadias, and cryptorchidism) harbored this *AR* mutation. It is noteworthy that these patients and the child we studied are all of Asian descent. In turn, the ExAC and gnomAD data indicate a relatively high prevalence of this *AR* variant in East Asia (1%‐1.5%) in the absence in other regions of the world (Table S2). Altogether, this might indicate the East Asian origin of this rare DSD‐related *AR* variant.

Alterations in *DHCR24* and *NODAL* are known to be associated with genital malformations (Table 1). *NODAL* (nodal growth differentiation factor) encodes a ligand of TGF‐beta (transforming growth factor‐beta) proteins, which regulate embryogenesis. Concerning DSD, *NODAL* is involved in the regulation of AR‐signaling pathways. In the DSD case presented here, we identified a *NODAL* variant (rs778607015) previously reported (ClinVar) in relation to Heterotaxy syndrome—a condition of the abnormal arrangement of the internal organs. The *DHCR24* variant (chr01:55349314:G>T) detected in the child has not been reported yet in public repositories. *DHCR24* (24‐dehydrocholesterol reductase) is involved in multiple pathways that produce cholesterol and, concerning DSD, is essential for steroid biosynthesis. Mutations in *DHCR24* have been found in association with desmosterolosis—a condition characterized by multiple congenital anomalies, including genital abnormalities.^20^ To note, in the studied case, a disturbance in steroid biosynthesis due to the *DHCR24* mutation may cause androgen insufficiency along with androgen insensitivity that may partly explain a positive effect of hormonal treatment in the child.

The remaining two candidate genes—*WDR48* (WD repeat domain 48) and *BMPR1B* (Bone morphogenetic protein receptor type 1B), has not been previously reported in association with DSD. Alterations in other members of the BMP signaling pathway—the *BMP4* and *BMP15* genes, are known to be related to DSD phenotypes.^9^ In addition, it is known that a *BMPR1B* paralog—*BMPR1A*, is involved in the process of regression of the Mullerian ducts, primordia of the oviducts, uterus, and upper vagina in male embryos. Our findings indicate that *BMPR1B* alterations may also be related to disturbances in the process of male embryo masculinization. *WDR48* encodes a regulator of deubiquitinating complexes and, consequently, has a broad range of functions. Mostly, *WDR48* alterations have been associated with neurodevelopmental abnormalities. However, *WDR48* also controls the development of male gonads, namely, the seminiferous tubule development. Here, we provide the first evidence that *WDR48* alterations may also be involved in DSD.

In conclusion, 46,XY DSD is a rare developmental disorder, highly heterogeneous both clinically and genetically. Consequently, it is possible that many disease‐causing mutations have yet not been identified. The results of our study demonstrate that WES provides a rapid and effective approach to identify novel mutations in DSD that may improve the diagnosis of the DSD patients.

## CONFLICT OF INTEREST

None declared.

## AUTHOR CONTRIBUTIONS

OYN and OVB: performed molecular analysis; OYN and SYR: performed genetic data analysis; MYS, MAZ, and IVO: performed the child's physical and behavior examination; IVP, IAA, and OVZ: collected medical records and clinical information and blood sample; ELG: conceptualized and coordinated this study; OYN: wrote the paper.

## ETHICAL APPROVAL

This study was approved by the Saint‐Petersburg State University Research Ethics Board. Informed written consent was obtained from the child's primary caregivers, orphanage officials. This report was prepared in accordance with the 1964 Helsinki Declaration and its later amendments or comparable ethical standards.

## Supporting information

Table S1

Table S2

Table S3
